# Evaluation of Resistance Induction Promoted by Bioactive Compounds of *Pseudomonas aeruginosa* LV Strain against Asian Soybean Rust

**DOI:** 10.3390/microorganisms12081576

**Published:** 2024-08-02

**Authors:** André Riedi Barazetti, Mickely Liuti Dealis, Kawany Roque Basso, Maria Clara Davis Silva, Leonardo da Cruz Alves, Maria Eugênia Alcântara Parra, Ane Stéfano Simionato, Martha Viviana Torres Cely, Arthur Ladeira Macedo, Denise Brentan Silva, Galdino Andrade

**Affiliations:** 1 Microbial Ecology Laboratory, Department of Microbiology, Universidade Estadual de Londrina, Londrina 86057-970, PR, Brazil; andrerbarazetti@gmail.com (A.R.B.); mickelyliuti@gmail.com (M.L.D.); kawbasso@gmail.com (K.R.B.); maria.clara.davis@uel.br (M.C.D.S.); leonardo.cruz.alves@uel.br (L.d.C.A.); maria.eugenia.alcantara@uel.br (M.E.A.P.); anessimionato@gmail.com (A.S.S.); 2 Agricultural and Environmental Sciences Institute, Federal University of Mato Grosso, Sinop 78550-728, MT, Brazil; vivianatorrescely95@gmail.com; 3 Natural Products and Mass Spectrometry Laboratory (LaPNEM), Federal University of Mato Grosso do Sul, Campo Grande 79070-900, MS, Brazil; arthur.ladeira@ufms.br (A.L.M.); denisebrentan@gmail.com (D.B.S.)

**Keywords:** antimicrobial, phenazine, metabolomic, flavonoid, *Phakopsora pachyrhizi*

## Abstract

*Pseudomonas* are known as higher producers of secondary metabolites with antimicrobial properties and plant growth promoters, including resistance induction. These mechanisms should be an alternative to pesticide use in crop production. *Phakopsora pachyrhizi* causes Asian soybean rust, representing a high loss of yield around the world. The objective of this paper was to evaluate the application of secondary metabolites produced by *Pseudomonas aeruginosa* LV strain from the semi-purified fraction F4A in soybean plants to induce plant resistance against *P. pachyrhizi* in field conditions. The experimental design was performed in randomized blocks with three replicates using two F4A doses (1 and 10 μg mL^−1^) combined or not with fungicides (Unizeb Gold^®^ or Sphere Max^®^). The control treatment, with Uni + Sph, saponins, flavonoids, and sphingolipids, showed higher intensities in the plants. In contrast, plants treated with the F4A fraction mainly exhibited fatty acid derivatives and some non-identified compounds with nitrogen. Plants treated with Sphere Max^®^, with or without F4A10, showed higher intensities of glycosylated flavonoids, such as kaempferol, luteolin, narigenin, and apigenin. Plants treated with F4A showed higher intensities of genistein and fatty acid derivatives. These increases in flavonoid compound biosynthesis and antioxidant properties probably contribute to the protection against reactive oxygen species (ROS).

## 1. Introduction

In the 2022/23 season, 78.5 million hectares were cultivated with soybean, and the yield was 322.8 million tons. Brazil is the one of biggest soybean producers and the fifth-largest nation in agricultural land area. In Brazil, the most widely grown crop is soybean (*Glycine max* L.), with 44 million ha sown and a yield of 154.6 million tons, followed by corn (*Zea mays* L.), with 22.6 million ha sown and a yield of 131.8 million tons in the 2022/23 season [1]. After thousands of years of use as an essential source of feed for humans and animals, soybean has emerged as one of the most significant crops on the global market and is the most important protein source for animal and human food [2].

There are some fungi causing soybean disease, such as anthracnose, stem canker, leaf blight, purple seed, downy mildew, powdery mildew, damping-off, and stem rot, and infections are caused by bacteria, viruses, and nematodes. However, none of them can match the destructive potential and economic impact of Asian soybean rust (ASR), caused by *P. pachyrhizi*. ASR should potentially yield losses of up to 90%, which corresponds to a billion-dollar loss on a global scale [3].

Urediniospore germination initiates the ASR infection process, which is subsequently followed by the development of germ tubes and appressoria [4,5]. The appressoria infect epidermal cells, and hypha grow through the intercellular spaces. The formation of haustoria in mesophyll cells, visible eruptions on the epidermis, and uredia occur around after 12 days, causing chlorosis and premature defoliation [6,7].

Some agricultural practices such as crop rotation, early-maturing cultivars, eliminating alternative plant hosts, and implementing sanitary voids are effective measures to mitigate the impacts of ASR. However, chemical control remains the most common and effective method to control *P. pachyrhizi*, preventing the secondary cycle of ASR and ensuring high productivity [8,9].

The Food and Agriculture Organization (FAO) [10] reported that in the year 2020, a significant amount of 2.7 million tons (Mt) of fungicide were used worldwide, resulting in the production of 7.2 Mt of formulated pesticide products valued at around USD 41.1 billion. The amount of fungicide used has increased 50% when compared with the levels in the 1990s. Brazil is the second largest consumer of pesticides, with a consumption of 377 kilotons (kt), closely behind the USA, which leads the use charts with 408 kt.

However, it is crucial to acknowledge that the use of pesticides carries potential risks of environmental contamination and for human and animal health. In addition, the exposure to pesticides can increase the emergence of pathogen-resistant microorganisms [11,12]. Fungal infections have been effectively controlled by site-specific fungicides like demethylation inhibitors (DMIs) and quinone outside inhibitors (QoIs) for years, but their efficiency has been decreasing [13,14].

Microorganisms such as fungi and bacteria produce several bioactive compounds, including alkaloids, steroids, terpenoids, peptides, polyketones, flavonoids, quinols, and phenols [15]. These compounds sometimes exhibit antioxidant, anti-inflammatory, and antibiotic properties. Additionally, endophytic bacteria can produce plant growth hormones, solubilize phosphate, assist in nutrient absorption, and fix N_2_ [15].

The challenge in agricultural research involves the identification of novel molecules, specifically bioactive compounds with antifungal activity, as these compounds show low environmental impact and low risks for human and animal health. In the last decade, innovative strategies for pest and disease management have emerged, emphasizing the use of compounds that trigger natural defense mechanisms in plants. These methods focus on mechanisms such as Inducing Systemic Resistance (ISR) or Systemic Acquired Resistance (SAR), which seek to improve plant defense response against pest and plant pathogens [16,17,18,19,20].

The *Pseudomonas* genera is found in the whole of the environment around the world. Some species are described as biocontrol agents, producing compounds such as phenazines, indolinones, peptides, glycopeptides, lipids, and aliphatic compounds with antimicrobial activity against fungi and bacteria pathogens for plants and humans [21,22,23,24].

Secondary metabolism compounds produced by *P. aeruginosa* LV strain were identified in the semi-purified fraction F4A as 1-carboxylic phenazine (CAP), with activity against *Botrytis cinerea* [25]; 1-carboxamide phenazine (CNP), with antimicrobial activity against *Staphyloccus aureus* [26]; and fluopsin C, a metaloantibiotic that controls *Xanthomonas citri* pv. citri [27] and *X. axonopodis* [28]. Also, the F4A fraction induced gene expression of β-1,3 glucanase in *Citrus sinensis* pv. In Valencia infected with *Cancidatus* Liberibacter asiaticus, the bacteria population decreased [29].

The objective of this paper was to evaluate the effect of the semi-purified fraction F4A produced by *P. aeruginosa* LV strain on SAR activation in soybeans infected with *P. pachyrhizi* by metabolomic analysis.

## 2. Materials and Methods

### 2.1. Production and Extract of Microbial Bioactive Compounds

*P. aeruginosa* strain LV was initially isolated from an old citrus canker lesion (*Citrus sinensis* cv. Valencia) in an orange leaf and is maintained in the collection of the Microbial Ecology Laboratory, Londrina, Brazil.

To produce bioactive compounds, *P. aeruginosa* LV strain was cultured in nutrient broth plus 5 mg L^−1^ CuCl_2_ for 10 days at 28 °C. After that, the culture was centrifuged at 9000 rpm for 15 min at 4 °C (Sorval RC-5C, Thermo Fisher Scientific, Waltham, MA, USA) and the supernatant was kept and the pellet discarded. The supernatant was reduced to 10% of its original volume in a chamber at 60 °C (SS Scientific, Londrina, Brazil). This concentrated sample was partitioned with dichloromethane (2:1, *v*/*v*) three times to obtain the dichloromethane phase (DP). DP was once concentrated with a rotatory evaporator (Büchi R-215, BUCHI Labortechnik, Flawil, Switzerland) at 45 °C at 100 rpm^−1^, and subsequently, it was submitted for vacuum liquid chromatography in a glass column (20 mm de diameter, 350 mm length) with silica gel 60 (0.063–0.200 mm, Merck, Darmstadt, Germany) as the stationary phase. The mobile phase applied (400 mL) was dichloromethane and ethyl acetate 1:1 (*v*/*v*) to obtain the fraction F4A [30].

### 2.2. Field Experiment

#### 2.2.1. Experimental Area

The experiments were carried out at the experimental farm of the State University of Londrina, PR, Brazil (−23.341223 S, −51.213271 W), and the soil was classified as Dystroferric Red Ferralsol [31]. Its chemical analysis was pH (CaCl_2_) 6.44, Al^3+^ 0.08 cmolc kg^−1^, Ca^2+^ 8.45 cmolc kg^−1^, Mg^2+^ 2.46 cmolc kg^−1^, K^+^ 0.15 cmolc kg^−1^, P 15.60 cmolc kg^−1^, and C 12.14 cmolc kg^−1^. The climate is Cfa, humid subtropical [32], with an average annual temperature of 24.2 °C, and pluviometry during the experiment from November to March was 69.88 L [33].

#### 2.2.2. Weed Control

Weed control was carried out with N (phosphonomethyl) glycine (Roundup WG, Monsanto do Brasil Ltda., Limoeiro, Brazil), and pest control was carried out with imidacloprid (Imida Gold 700 WG, UPL, Ituverava, Brazil), Thiamethoxam + Lambda-Cyalothrin (Engeo Pleno^TM^ S, Syngenta, São Paulo, Brazil), and Spiromesifen (Oberon, Bayer S.A., São Paulo, Brazil) according to the manufacturers’ instructions.

#### 2.2.3. Effect of F4A Fraction on Asian Soybean Rust

The experimental design was randomized from six treatments and three replicates and evaluated two concentrations of F4A (1 and 10 μg mL^−1^) added or not to Unizeb Gold^®^ (Uni) (UPL, Ituverava, Brazil) and/or Sphere Max^®^ (Sph) (Bayer S.A., São Paulo, Brazil), according to the manufacturers’ instructions, to evaluated the following treatments: F4A1, F4A1 + Uni, F4A1 + Sph, F4A10, F4A10 + Uni, and F4A10 + Sph. The positive control (Unizeb Gold^®^ and Sphere Max^®^) was an additional group named Uni + Sph. The parcel had a total area of 9 m^2^ and ten lines.

Seeds of soybean var. NS 5959 IPRO (Nidera Seeds, São Paulo, Brazil) were treated with Metalaxyl-M, Fludioxonil (Maxim^®^ XL, Syngenta, São Paulo, Brazil), and Imidachloprid + Thiodicarb (CropStar^®^, Bayer S.A, São Paulo, Brazil). Soil was fertilized with 250 kg ha^−1^ of NPK 10-15-15, and seeds were inoculated with Rizo Plus^®^ (Rhizobacter, Londrina, Brazil) (*Bradyrhizobium japonocum* strain SEMIA 5079 and SEMIA 5080) according to the manufacturer’s instructions and Arbuscular mycorrhiza fungi *R. clarus* at a concentration of around 47.5 spores per gram of seeds [34].

The F4A fraction and pesticides were applied with a backpack sprayer with a CO_2_ cylinder (Agro Pesquisa, Campinas, Brazil) with a flow of 200 L ha^−1^. The application was carried out two times (A1 and A2). A1 was carried out in R5.1, right after the first symptoms of Asian soybean rust, and A2 was carried out in R6. After 24 h for each application, five leaves were collected from each parcel and frozen in dry ice.

### 2.3. Metabolomic Analysis

#### 2.3.1. Sample Preparation

From the five leaves collected in the field, the healthy leaves were selected, washed, and powdered by liquid nitrogen in a pistil. From these materials, 0.2 g was extracted with 10 mL methanol and water 8:2 (*v*/*v*) in an ultrasonic bath (30 min) (Branson 1510-DTH, Marshall Scientific, Hampton, NH, USA); subsequently, the samples were centrifuged for 10 min at 6.000 rpm and 8 °C (Jouan CR3i, Thermo Fisher Scientific, Waltham, MA, USA). The supernatants were filtered by Millex syringe filters (PTFE, 0.22 mm × 13 mm, Merck Millipore, Burlington, MA, USA) and added to vials of 1.5 mL to carry out the chromatography analysis. Aliquots of 20 µL of each sample were pooled to prepare the quality control sample (QC).

#### 2.3.2. Metabolomic Analysis

The metabolomic data analysis was carried out in an Ultra-Fast Liquid Chromatograph LC-20AD Shimadzu Prominence (Shimadzu, Kyoto, Japan) coupled to a diode array detector and mass spectrophotometer with ionization source electrospray and analyzer quadrupole and time of flight (MicrOTOF-Q III Bruker Daltonics, Billerica, MA, USA).

The samples (1 µL) were processed in the chromatographic system using a Kinetex C18 column (2.6 μm, 100 A, 150 × 2.1 mm, Phenomenex, Torrance, CA, USA). The mobile phase was composed of acetonitrile (B) and water (A), with formic acid 0.1% (*v*/*v*) added to both phases. The gradient elution profile was the following: 0–2 min 3% B, 2–25 min 3 to 25% B, 25–40 min 25 to 80% B, and 40–43 min 80% B. The flow rate was 0.3 mL min^−1^, and the chromatographic column was maintained at 50 °C during the analysis. Samples were analyzed in positive and negative ion mode. Nitrogen was used as a nebulizer (4 Bar) and drier (9 L/min) and collision gas. The compound annotation was based on the spectral data from UV, MS, and the fragmentation profile compared with the databases and data reported in the literature [35,36,37]. Some compound data were confirmed by the co-injection of authentic standards.

The data were initially analyzed by Data Analysis 4.2 software (Bruker Daltomics, Billerica, MA, USA)) and were subsequently aligned and reduced by the software Metalign (version 011012) and MSClust (version 300817), respectively. The statistical analyses were performed by the platform Metaboanalyst 5.0, and the data were previously normalized by median, log transformed, and auto-scaled before these analyses. The statistical analyses include hierarchical cluster heatmap (HCH) and principal component analysis (PCA).

## 3. Results

The dataset was composed of 129 entrances after data processing. The spectral data and annotated compounds from selected entrances obtained by statistical analyses are summarized in Table 1.

**Figure 1:**
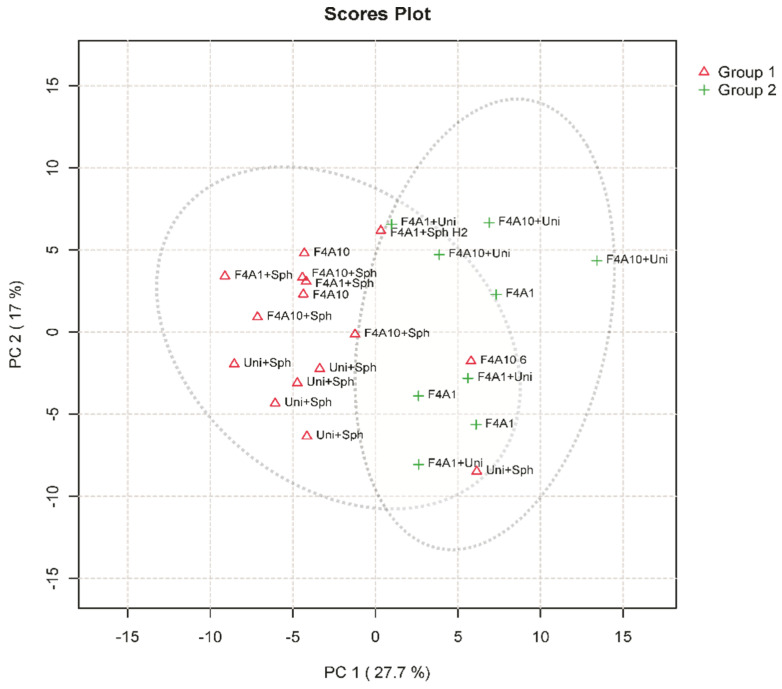
Principal component analysis (PCA) score plot from soybean leaf samples infected with *Phakopsora pachyrhizi* under different treatments. Group 1, represented by red triangles, was formed by Uni + Sph, F4A1 + Sph, F4A10 + Sph, and F4A10. Group 2, represented by green plus symbols, was formed by F4A1, F4A1 + Uni and F4A10 + Uni.

Figure 1 presents the principal component analysis (PCA) score plot from the LC-MS data of soybean leaf samples infected with *Phakopsora pachyrhizi* under different treatments. PCA is an unsupervised statistical technique that transforms high-dimensional data into a lower-dimensional coordinate system, preserving the highest variance in the data.

In the PCA analysis, group 1, represented by red triangles, includes the treatments Uni + Sph F4A1 + Sph, F4A10 + Sph, and F4A10, while group 2, represented by green plus symbols, comprises the treatments F4A1, F4A1 + Uni, and F4A10 + Uni. The two groups show a tendency of separation between the groups (Figure 1).

**Figure 2:**
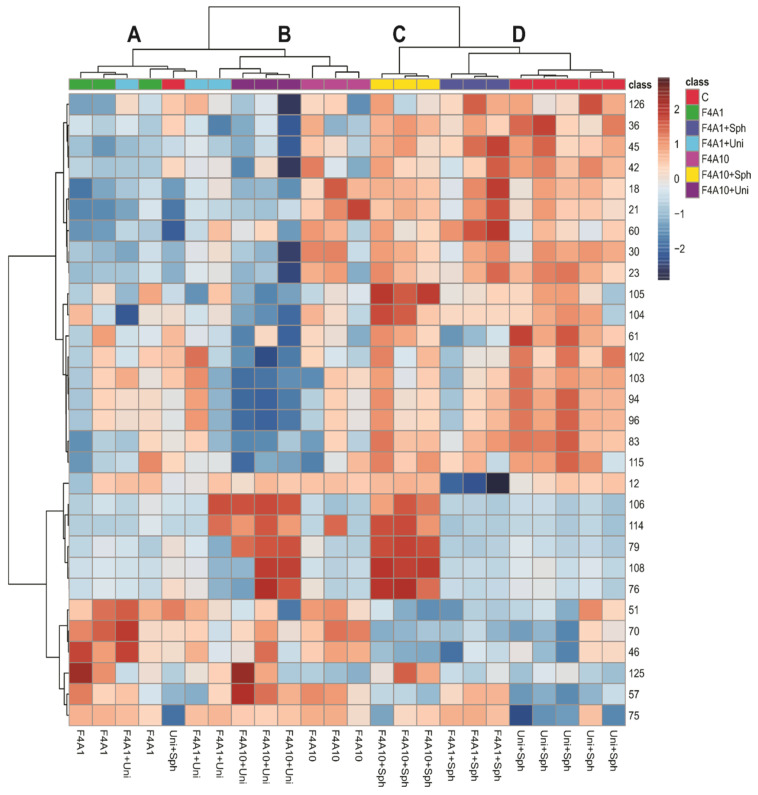
Heatmap and hierarchical clustering analysis (HCA) of the soybean leaf samples infected with *Phakopsora pachyrhizi* under different treatments. Treatments are control (C), F4A1, FAA1 + Sph, F4A1 + Uni, F4A10, F4A10 + Sph, and F4A10 + Uni. The clusters A–D were highlighted in the HCA.

Figure 2 shows a heatmap and hierarchical clustering analysis (HCA) of soybean leaf samples infected with *P. pachyrhizi* under different treatments. The heatmap visualizes the relative intensity of specific compounds in the samples, where darker red colors indicate higher intensity. The HCA groups the samples based on the similarities in their metabolic profiles, highlighting four main clusters: A, B, C, and D.

The clusters C and D and the group F4A10 exhibited higher intensities of compounds such as *O*-di-hexosyl kaempferol (18), *O*-di-hexosyl luteolin (21), *O*-di-deoxyhexosyl hexosyl kaempferol (23), *O*-hexosyl kaempferol (30), *O*-hexosyl malonyl naringenin (45), apigenin (60), and compounds 36, 42, and 126. These compounds are glycosylated flavonoids, suggesting that the use of Sphere Max^®^ induces the accumulation of these glycosylated flavonoids, which may be related to increased plant resistance against *P. pachyrhizi*. Additionally, the samples treated with F4A1, FaA1 + Uni, F4A10, and F4A10 + Uni are more similar (cluster A and B), while the treatments with F4A1 + Sph are more similar to the control.

**Figure 3:**
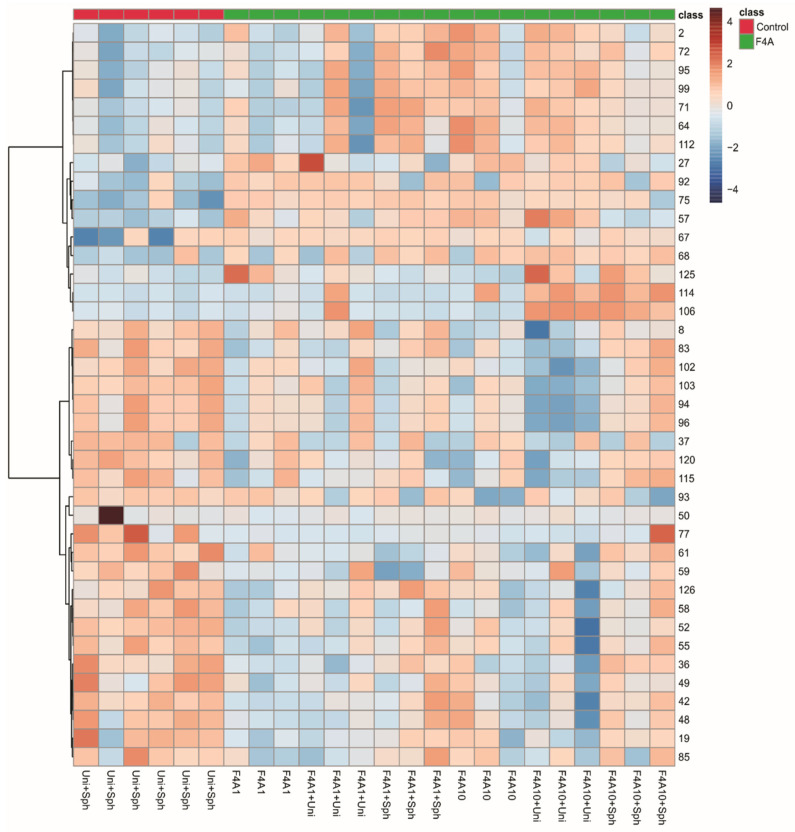
Heatmap of the soybean leaf samples infected with *Phakopsora pachyrhizi* from control (red) and the fraction F4A1 (green) treatments.

Since the clustering did not show a pattern that could be induced by treatment with F4A, we decided to observe the heatmap grouping the samples as control and treatment with fractions F4A and with the most important 40 peaks (Figure 3). These results indicated higher intensities of O-hexosyl genistein (27), genistein (57), two fatty acid derivatives (125 and 112), and peaks 2, 64, 67, 68, 71, 72, 75, 92, 95, and 99 in all treatments and peaks 106 and 114 in treatments with F4A10 (Figure 2). The samples treated with Uni + Sph show higher intensities of some compounds, such as *O*-di-deoxyhexosyl kaempferol (19), *O*-hexosyl-malonyl apigenin (48), *O*-hexosyl-malonyl genistein (49), *O*-hexosyl malonyl afrormosin (59), sphingolipid (77), and saponin (85) (Figure 3).

The compounds with higher intensities in the control samples (Uni + Sph) were different from the induced metabolites for the plant treated with the fraction F4A (different combinations evaluated). The saponins (85, 94, 96, 103), *O*-glycosyl-malonylated flavonoids (36, 48, 49, 59), and the sphingolipid 77 showed higher intensities in the plants treated with Uni + Sph, while in the plants treated with the fraction F4A were mainly fatty acid derivatives (112, 125) and some non-identified compounds with nitrogen (67–68, 71, 75, 92, 106, 114).

The annotated compounds included flavonols, flavones, isoflavones, saponins, and fatty acid derivatives. The most annotated flavonoids were *O*-glycosides, and the UV data were useful to determine the flavonoidic classes, such as the flavonols (λmax ≈ 268 and 350 nm; peaks 15, 17–19, 23, 24, 29, 30, and 33–34), isoflavones (λmax ≈ 270 and 330/shoulder nm; peaks 13, 25, 27, 36, 40, 43, 49, 53, 56, and 59), and flavones (λmax ≈ 268 and 335 nm; peaks 21, 26, 38-39, 48, and 60). The determination of the molecular formulas was based on accurate mass considering the errors and mSigma up to 8 ppm and 30 ppm, respectively. The losses of 162, 146, 176, and 86 *u* suggested the hexosyl, deoxyhexosyl, glucuronyl, and malonyl substituents bound with *O*-linked type. For example, the compounds 40 and 43 revealed the fragment ions at *m*/*z* 271 relative to the aglycone genistein, which were yielded from the losses of 248 *u* compatible to losses of the hexosyl (162 *u*) and malonyl (86 *u*) groups [38]. Thus, several compounds were annotated from the samples.

## 4. Discussion

Unizeb Gold^®^ is a multi-site fungicide. Its active ingredient is mancozeb, which belongs to the group of dithiocarbamates. When exposed to water, mancozeb breaks down and releases ethylene bisisothiocyanate sulfide (EBIS). This compound is then converted into ethylene bisisothiocyanate (EBI) by UV light. The mode of action is not fully elucidated; however, EBIS and EBI are believed to be toxic to enzymes containing sulfhydryl groups [39].

Sphere Max^®^ (Bayer Co.), in turn, is a systemic fungicide containing cyproconazole and trifloxystrobin, which belongs to the groups of triazoles and strobilurins, respectively. Triazoles inhibit cytochrome P450 14α-demethylase, directly interfering in lanosterol-to-ergosterol conversion and affecting cell membrane formation, which causes disruption of cells [40,41]. Also, triazoles inhibit spore germination, appressorium formation, and mycelium growth in the host tissue [42,43]. On the other hand, strobilurins act by inhibiting the respiratory chain [44].

Those effects were observed in a field experiment on soybeans [45]. The treatments Uni + Sph, F4A1 + Sph, and F4A10 + Sph were highly effective in controlling ASR, delaying disease progression, and causing damage to the spores and hyphae of *P. pachyrhizi*. In the same study, Barazetti et al. [45] observed in an in vitro experiment that F4A1 and F4A10 inhibit spore germination and germinative tube growth.

Soybean treated with Sphere Max^®^ increased production of flavonoids, which suggested that Sphere Max^®^ induces plant defense. Strobilurins have been related as inducing priming responses in *Arabidopsis* [46], and the same results were observed in *Nicotiana tabacum* treated with pyraclostrobin, decreasing tobacco mosaic virus [47], and in cucumber treated with the same compound against mosaic virus and *P. syringae* pv. *tomato* [48].

Despite the resistance inducing effects, there is no data in the literature that associate the application of the active ingredients present in Unizeb Gold^®^ and Sphere Max^®^ with the biosynthesis of flavonoids as observed in our investigation.

Plants treated with Uni + Sph also exhibit higher levels of saponins. This one is part of the terpene class, mainly present in dicotyledonous species, and is related to insect and fungal attack in plants. In both cases, this activity is commonly linked to their interaction with biological membranes, forming complexes with sterols, proteins, or phospholipids. More recently, it was verified that saponins also have the potential to activate defense responses through salicylic acid induction and oxidative burst in *Brassica napus* against *Leptosphaeria maculans* [49]. Sadly, the interaction between saponin levels and *P. pachyrhizi* infection on soy plants is still unclear.

The F4A fraction obtained from the *P. aeruginosa* LV strain contains two phenazines, compounds that have been reported as biocontrol agents through antagonism to other microorganisms in the rhizosphere besides being important to control fungal crop diseases [50]. In general, phenazines, especially pyocyanin, promote the generation of ROS, which act as signaling molecules involved in growth processes, development, and defense responses against pathogens through ISR [51].

The inoculation of *P. aeruginosa* in rice stimulated plant defense against *Magneporthe grisea*, which causes blast disease, and decreased infection. Pyocyanin (PYO) was crucial as an elicitor of ISR. When rice was treated with 25 ηM, 1 ηM, and 100 ηM of PYO, the infection of *M. grisea* was reduced, but no effect was observed with *Rhizoctonia solani* [52]. The ISR should explain the effect observed on *P. pachyrhizi* in our experiment, where soybeans treated with 1 µg F4A increased soybean yield to 4.64 ton ha^−1^, 6.2% more than plants treated with Uni + Sph. Another study demonstrated that F4A activates soybean defenses, increasing the expression of phenylalanine ammonia lyase (PAL), O-methyltransferase (OMT), and pathogenesis-related protein-2 (PR-2; glucanases) defense-related genes, detected 24 and 72 h after soybean sprouts were sprayed with purified Fluopsin C, which is one of the compounds that contains the F4A fraction [53].

The enzyme PAL converts phenylalanine to trans-cinnamic acid and ammonia; the first one should be incorporated into many phenolic compounds and is present in esters, coumarins, lignin, and flavonoid formation [54]. It is largely known that flavonoids have antioxidant properties due to the presence of B-ring catechol groups and other factors which donate electron-reducing ROS [55]. The production of phytoalexin glyceollin is one of the most common responses of soybeans against phytopathogenic fungi. Silva et al. [54], studying soybean leaves inoculated and not inoculated with *P. pachyrhizi*, observed that this phytoalexin was only expressed in the second group. The metabolic precursor of glyceollin is daidzein, which is present in soybean plants treated with F4A. Both glyceollin and daidzein have antioxidant activity against ROS [56,57,58], as well as other compounds of the flavonoid group that were identified in plants treated with F4A, such as kaempferol [59,60], apigenin [61], and luteolin [55], and should be related to the plant protection against *P. pachyrhizi* observed in our results. Gupta et al. [62], studying *Arabidopsis thaliana* plants treated with ethylene, a hormone known to be involved in stress responses, observed that there was an increase in isoflavonoid accumulation when compared to control plants, especially genistin (2.7 fold), daidzein (21.38 fold), and genistein (7.6 fold). They also verified an increment in levels of fatty acids and flavonoids, suggesting an important role of these compounds in plant defense besides being part of the ethylene pathway.

Plants treated with F4A10 + Sph increased sphingolipid and pheophorbide synthesis. Sphingolipids are important for cell membrane structure, cell-to-cell signalization, cell wall formation, stomatal closure, and controlled cell death [63,64]. Pheophorbide A is derived from chlorophyl degradation during the natural process of leaf senescence after seed formation [65] or by biotic and abiotic stress or fungal infection as in *P. pachyrhizi* [7].

In general, all treatments showed the presence of fatty acids or their derivates, such as octadecatrienoic acid and eicosatetraenoic acid, which were probably related to the grain formation of soybeans during our first (R5) and second (R6) harvests. Once stored, seeds that have these lipids as their main reserve form are subjected to slow and consistent exposure to oxygen, forming hydroperoxides, other oxygenated acids, and free radicals [66].

Lipids and fatty acids can also act in defense responses to biotic and abiotic stresses. Linolenic and linoleic acids, for example, are precursors of oxylipins in plants, a product of self-oxidation or enzymatic oxidation [54].

Through the heatmaps, it is also possible to observe some variations in the production of compounds, even within the same treatments. This probably stems from the fact that the experiment was conducted under field conditions, where a series of biotic and abiotic factors, including temperature, humidity, wind, pests, and weeds, cannot be fully controlled [67].

In conclusion, our results suggest that the F4A fraction induced plant resistance against Asian soybean rust, decreasing lesion formation and increasing soybean yield.

## Figures and Tables

**Table 1:** Annotated compounds from samples of controls and treatments with F4A.

Peak	RT (min)	Compound	UV (nm)	MF	MS (*m*/*z*) [M + H]^+^	MS/MS
13	16.03	*O*-hexosyl daidzein	272, 330 ^sh^	C_21_H_20_O_9_	417.1169	255, 227, 199
15	17.06	*O*-deoxyhexosyl di-hexosyl kaempferol	265, 348	C_33_H_40_O_20_	757.2171	287
17	17.40	*O*-deoxyhexosyl di-hexosyl kaempferol	265, 346	C_33_H_40_O_20_	757.2163	287
18	17.71	*O*-di-hexosyl kaempferol	265, 345	C_27_H_30_O_16_	611.1602	449, 287
19	17.75	*O*-di-deoxyhexosyl kaempferol	265, 345	C_21_H_20_O_11_	449.1088	271
21	18.00	*O*-di-hexosyl luteolin	268, 335	C_27_H_30_O_16_	611.1607	287
22	18.25	NI	280	C_13_H_20_O_2_	209.1529	-
23	18.35	*O*-di-deoxyhexosyl hexosyl kaempferol	265, 347	C_33_H_40_O_19_	741.2251	595, 449, 287
24	18.72	*O*-di-deoxyhexosyl hexosyl kaempferol	265, 348	C_33_H_40_O_19_	741.2245	595, 449, 287
25	19.03	*O*-hexosyl-malonyl daidzein	275, 330 ^sh^	C_24_H_22_O_12_	503.1203	255
26	19.11	*O*-deoxyhexosyl hexosyl luteolin	266, 338	C_27_H_30_O_15_	595.1678	449, 287
27	19.21	*O*-hexosyl genistein	260, 330 ^sh^	C_21_H_20_O_10_	433.1139	271
29	19.75	*O*-deoxyhexosyl hexosyl kaempferol	265, 348	C_27_H_30_O_15_	595.1655	449, 287, 153
30	20.28	*O*-hexosyl kaempferol	265, 347	C_21_H_20_O_11_	449.1073	287
33	20.76	*O*-deoxyhexosyl hexosyl kaempferol	265, 349	C_27_H_30_O_15_	595.1650	449, 287, 241, 165
34	21.09	*O*-hexosyl kaempferol	265, 349	C_21_H_20_O_11_	449.1090	287
36	21.35	*O*-hexosyl-malonyl glycitin	270, 330 ^sh^	C_25_H_24_O_13_	533.1274	371, 285
38	21.65	*O*-hexosyl apigenin	268, 335	C_21_H_20_O_10_	433.1147	271
39	21.85	*O*-pentosyl luteolin	265, 332	C_20_H_18_O_10_	419.0981	287
40	22.20	*O*-hexosyl-malonyl genistein	275, 330 ^sh^	C_24_H_22_O_13_	519.1113	271
42	23.38	NI	-	C_22_H_32_O_10_	457.2076	439, 191, 173
43	23.48	*O*-hexosyl-malonyl genistein	259,330 ^sh^	C_24_H_22_O_13_	519.1131	271
44	23.79	NI	-	C_22_H_32_O_10_	457.2061	439, 191, 173
45	23.85	*O*-hexosyl-malonyl naringenin	280, 330 ^sh^	C_24_H_24_O_13_	521.1286	273, 153
48	24.99	*O*-hexosyl-malonyl apigenin	267, 334	C_24_H_22_O_13_	519.1126	271
49	25.40	*O*-hexosyl-malonyl genistein	275, 330 ^sh^	C_24_H_22_O_13_	519.1131	271
53	27.19	*O*-hexosyl-malonyl afrormosin	278, 330 ^sh^	C_26_H_26_O_13_	547.1498	299
56	27.96	*O*-hexosyl-malonyl afrormosin	257, 320 ^sh^	C_26_H_26_O_13_	547.1478	299
57	28.52	Genistein	278, 330 ^sh^	C_15_H_10_O_5_	271.0611	271, 215
59	29.04	*O*-hexosyl-malonyl afrormosin	280, 328 ^sh^	C_26_H_26_O_13_	547.1489	299
60	29.35	Apigenin	267, 335	C_15_H_10_O_5_	271.0609	271, 243, 229, 153
64	30.78	NI	-	C_20_H_30_O_5_	351.2153	275
67	32.52	NI	-	C_18_H_23_NO_2_	286.1785	-
68	32.68	NI	-	C_18_H_25_NO_2_	288.1956	270, 172, 159
71	32.91	NI	280	C_10_H_18_N_2_O_5_	247.1300	-
72	33.05	Saponin	-	C_51_H_80_O_21_	1029.5243	
76	33.22	NI	280, 320	C_16_H_21_NO	244.1693	172, 159
77	33.36	Sphingolipid (phytosphingosine isomer)	-	C_18_H_39_NO_3_	318.2995	256
82	33.66	Saponin (soyaponin ag isomer)	-	C_54_H_84_O_22_	1085.5510	599, 581, 563, 463, 405, 365, 217, 203
85	33.72	Saponin	-	C_51_H_80_O_21_	1029.5256	
86	33.87	Soyasaponin I	-	C_48_H_78_O_18_	943.5258	599, 581, 441, 423, 405, 383, 365, 315, 247, 217, 203
89	34.25	Saponin		C_51_H_80_O_21_	1029.5272	
91	34.50	Saponin	-	C_55_H_72_O_13_	941.5099	597, 439, 421, 410, 381, 313, 273, 245, 219
94	34.84	Saponin (soyasaponin βg)	-	C_54_H_84_O_21_	1069.5546	923, 761, 725, 599, 581, 567, 441, 423, 217
95	34.87	NI	-	C_20_H_28_O_4_	333.2047	-
96	34.87	Saponin (soyasaponin βg)	-	C_54_H_84_O_21_	1069.5546	923, 761, 725, 599, 581, 567, 441, 423, 217
100	35.26	Pterocarpan	280, 325 ^sh^	C_21_H_20_O_4_	337.1430	279, 267
103	35.46	Saponin	-	C_48_H_74_O_17_	923.4965	-
105	35.89	Fatty acid derivative	-	C_21_H_36_O_4_	353.2691	-
111	36.78	Fatty acid derivative	-	C_18_H_28_O_2_	277.2164	-
112	36.91	Fatty acid derivative	-	C_18_H_28_O_2_	277.2152	-
115	37.33	Fatty acid derivative	-	C_21_H_36_O_4_	353.2691	-
119	40.84	Eicosatetraenoic acid	-	C_20_H_32_O_2_	305.2496	-
122	41.38	Octadecatrienoic acid	-	C_18_H_30_O_2_	279.2295	-
125	42.34	Fatty acid derivative	-	C_21_H_36_O_4_	353.2694	-
129	43.34	Pheophorbide A	280, 410	C_35_H_36_N_4_O_5_	593.2751	565, 533, 461

RT: retention time, ^sh^: shoulder.

## Data Availability

The original contributions presented in the study are included in the article, further inquiries can be directed to the corresponding author.
